# The Nuclear Damage-Response Code of Cutaneous Wound Repair: Mechanotransduction, cGAS-STING, Senescence, and Regenerative Interfaces

**DOI:** 10.3390/biom16101427

**Published:** 2026-10-01

**Authors:** Muhammad Saeed Akhtar, Mohammad Aslam, Mirza Masroor Ali Beg

**Affiliations:** 1 Department of Chemistry, Yeungnam University, Gyeongsan 38541, Republic of Korea; 2 Department of Chemical Engineering, Yeungnam University, Gyeongsan 38541, Republic of Korea; mohammadaslam13@yu.ac.kr; 3 Faculty of Medicine, Ala-Too International University, Bishkek 720048, Kyrgyzstan; mirzamasroor.alibeg@alatoo.edu.kg

**Keywords:** wound healing, chronic wounds, mechanotransduction, nuclear envelope, cGAS-STING, senescence, diabetic foot ulcer, biomaterials

## Abstract

Chronic cutaneous wounds are often described as disorders of inflammation, infection, perfusion, or extracellular matrix turnover, yet these categories do not fully explain why a wound that initially detects injury fails to re-enter a coordinated program of repair. This Perspective proposes a unifying model in which the nucleus acts as a damage integrator at the wound edge. Mechanical strain, osmotic swelling, oxidative stress, chromatin fragmentation, and mitochondrial injury converge on nuclear-envelope tension, nuclear rupture, cytoplasmic deoxyribonucleic acid sensing, and senescence-associated transcription. When the signal is brief and spatially restricted, it can support leukocyte recruitment, keratinocyte migration, matrix remodeling, and early host defense. When it is amplified by diabetes, aging, tissue stiffness, biofilm-compatible inflammation, or defective autophagic and efferocytic clearance, the same pathway becomes a self-reinforcing loop that sustains interferon signaling, inflammasome activity, endothelial dysfunction, fibroblast memory, and impaired epidermal differentiation. The review integrates evidence from single-cell wound atlases, mechanobiology, innate immune deoxyribonucleic acid sensing, mitochondrial stress, macrophage biology, senescence, microbiome studies, and smart biomaterials. It argues that future wound therapeutics should not simply accelerate closure, but should classify and reset the temporal polarity of nuclear damage responses. Nucleus-aware dressings, local signal-gated inhibitors, senolytic or senomorphic interfaces, and biomaterials that reduce pathological mechanical memory may provide more precise ways to convert chronic wound fields into regenerative ones.

## 1. Introduction

Chronic cutaneous wounds remain a global clinical problem because they combine tissue loss with persistent biological misclassification: the wound continues to behave as if acute danger is unresolved even after the original insult has passed. Estimates of wound prevalence and cost show that chronic wounds are not a marginal complication but a major health-system burden, with recurrent diabetic foot ulcers, venous leg ulcers, and pressure injuries consuming large resources and quality-adjusted life years [1,2,3]. Diabetic foot ulcer recurrence is especially important because closure alone does not restore durable tissue competence; recurrence rates and limb-threatening complications indicate that the local tissue often remains metabolically, immunologically, and mechanically primed for breakdown [4]. Conventional frameworks emphasize ischemia, neuropathy, bacterial burden, excess protease activity, and impaired growth-factor signaling, and these remain clinically indispensable explanations [5]. Recent biomaterial studies further support this background by showing that wound dressings are moving from passive coverage toward diagnostic, microenvironment-responsive, antibacterial, and healing-evaluation platforms; nanomaterial-based dressings can integrate sensing and therapeutic functions, and dendritic antibacterial hydrogels can reduce bacterial burden while supporting hemostasis, macrophage phenotype transition, collagen deposition, and vascular formation in infected wound models [6,7]. However, these categories do not fully explain why chronic wounds repeatedly re-enter inflammation after debridement, why some histologically similar wounds heal and others stall, or why therapies that accelerate area reduction often fail to rebuild tissue resilient enough to avoid recurrence.

The canonical sequence of hemostasis, inflammation, proliferation, and remodeling is useful only if it is treated as a dynamic network rather than a timetable. Cellular reviews of wound repair show that neutrophils, macrophages, keratinocytes, endothelial cells, fibroblasts, pericytes, nerves, and matrix all change function according to local timing and spatial context [8,9]. A short inflammatory burst can clear debris and initiate repair, whereas metabolically sustained inflammation drives excess reactive oxygen species, protease activity, and fibroblast dysfunction [10]. Single-cell wound studies have added a crucial layer: cells in wounds do not occupy fixed identities, but form context-dependent states that differ across anatomical sites, diabetic status, healing trajectory, and injury stage [11,12,13]. Human diabetic foot ulcer atlases further show that non-healing is not simply a lack of repair cells; it is a disordered ecosystem with altered fibroblast, macrophage, endothelial, keratinocyte, and immune-cell states [14,15,16].

A growing body of regenerative biology indicates that scarring and regeneration diverge through fibroblast lineage, developmental memory, and injury-induced plasticity. Cross-tissue fibroblast maps support the idea that fibroblasts are organized into conserved functional programs rather than a single stromal population [17]. Multi-omic comparisons of scarring and regenerative wound models reveal distinct molecular events before visible scar outcomes appear, which implies that repair fate is encoded early at the level of cell state, matrix interaction, and inflammatory tone [18]. Engrailed-1-positive fibroblast activation, adult dermal Lef1 expression, fibroblast regenerative programs, hair-follicle neogenesis, adipocyte regeneration from myofibroblasts, and injury-site myeloid-to-fibroblast-like conversion all show that adult skin retains latent plasticity under specific conditions [19,20,21,22,23,24]. These studies justify a review that focuses on upstream control logic rather than another inventory of individual growth factors or dressings.

The central aim of this Perspective is to define the nuclear damage-response code as an underused framework for explaining why cutaneous wounds either resolve, scar, or become chronic. The specific objectives are to synthesize how mechanical forces and nuclear-envelope stress transmit injury information to chromatin; to connect cytoplasmic DNA sensing and cGAS-STING signaling with mitochondrial damage, senescence, macrophage polarization, endothelial dysfunction, and keratinocyte differentiation; to identify temporal rules that separate adaptive from pathogenic nuclear stress signaling; and to propose testable biomaterial and clinical designs that reset this code without suppressing useful early defense.

The distinctive contribution of this Perspective is not the description of any single wound pathway. Its novelty lies in treating nuclear deformation, chromatin leakage, mitochondrial DNA release, cGAS-STING signaling, senescence, efferocytosis, extracellular chromatin, and adaptive biomaterial design as one time-dependent decision system. To avoid overextension, the review separates direct wound evidence from mechanistic evidence imported from adjacent fields and uses the latter to generate testable hypotheses rather than to claim established clinical causality.

## 2. Search Strategy and Scope

This narrative Perspective was scoped to recent mechanistic literature in cutaneous wound healing, nuclear mechanotransduction, cytosolic DNA sensing, mitochondrial inflammatory signaling, senescence, macrophage and neutrophil biology, wound microbiology, fibrosis, and regenerative biomaterials. Because the article is not a systematic review, it does not make pooled effect estimates or grade clinical interventions. Instead, papers were prioritized when they directly linked a molecular mechanism to wound-relevant cell behavior, provided human wound or single-cell evidence, or offered a translational intervention that could be interpreted through the nuclear damage-response model. Foundational older studies were retained only when newer articles depended on their mechanistic principles, such as fibroblast lineage mapping, integrin mechanotransduction, and nuclear lamina mechanics [25,26,27,28]. Recent studies were weighted heavily when they added cell-state specificity or disease relevance, especially for diabetic wounds and scar-forming fibroblast programs [15,16,18,19].

Searches were performed in PubMed/MEDLINE, Web of Science, Scopus, Google Scholar, and publisher databases including Elsevier ScienceDirect, Springer Nature, Wiley, ACS Publications, MDPI, Frontiers, and Oxford Academic. The main search window was January 2013 to September 2026, with priority given to articles published from 2018 onward unless an older work provided a foundational mechanism. Keyword combinations included “cutaneous wound healing”, “diabetic foot ulcer”, “chronic wound”, “single-cell RNA sequencing”, “fibroblast heterogeneity”, “mechanotransduction”, “nuclear envelope rupture”, “lamin A/C”, “cGAS-STING”, “cytosolic DNA sensing”, “mitochondrial DNA”, “senescence”, “SASP”, “neutrophil extracellular traps”, “efferocytosis”, “biofilm”, “smart dressing”, “hydrogel”, “biomaterial”, and “wound biomarker”.

Evidence was interpreted hierarchically. Direct human wound and animal wound-healing studies were treated as the strongest anchors for disease relevance; single-cell, transcriptomic, biomaterial, and microbiome studies were used to assign cell states and translational readouts; mechanobiology, cancer-cell migration, senescence, and mitochondrial immunology studies were used only where they defined mechanisms that can be tested in wounded skin. Claims based on this third category are framed as hypotheses or plausible sequences, not as proven wound biology.

The review deliberately treats mechanotransduction and inflammation as coupled rather than separate axes. In fibroblast-rich matrices, collagen organization, contractile force, macrophage attraction, and myofibroblast heterogeneity form feedback loops that can either restore tissue architecture or lock a wound into fibrotic memory [29,30,31]. At the chromatin level, mechanical inputs can alter gene silencing, lineage commitment, and mechanosensitive transcriptional availability [32]. Nuclear lamin abundance scales with matrix stiffness and cellular differentiation in multiple tissues, making the nucleus a plausible memory device for wound stiffness rather than a passive container [33]. These considerations define the scope of the article: the nuclear damage-response code is not proposed as a single pathway but as a convergent control layer that integrates matrix force, cytoskeletal continuity, chromatin exposure, mitochondrial stress, and inflammatory clearance (Table 1).

## 3. Nuclear Mechanics as a Wound-Sensing Axis

The wound edge is mechanically unusual because cells suddenly encounter a free boundary, disrupted basement membrane, edema, altered osmotic pressure, exposed matrix ligands, and tensile gradients from surrounding skin. The nucleus is well positioned to integrate these cues because it is physically coupled to the cytoskeleton and extracellular matrix through actin, intermediate filaments, microtubules, linker-of-nucleoskeleton-and-cytoskeleton complexes, and lamins [34,35]. Recent work shows that the nucleus can measure shape changes and spatial confinement, adjusting cell behavior when cytoplasmic dimensions and nuclear deformation diverge [36,37]. In a wound, this sensing function is not merely a migration problem. Keratinocytes, fibroblasts, endothelial cells, and immune cells must all pass through compressed or reorganizing environments, and each cell type can convert nuclear strain into transcriptional, repair, or inflammatory outputs. The important mechanistic point is temporal: nuclear deformation may provide useful positional information during early closure, but repeated or unresolved deformation can convert positional sensing into damage signaling.

Tissue injury can convert nuclear mechanics into inflammation even without microbial invasion. A zebrafish epithelial injury model showed that the cell nucleus can act as a mechanotransducer of tissue damage-induced inflammation, with nuclear-associated signaling contributing to leukocyte recruitment [38]. In mammalian cells forced through tight spaces, nuclear-envelope rupture exposes nuclear contents, creates DNA damage, and requires membrane repair systems; these mechanisms were first characterized in migratory cancer cells but are directly relevant to wound-edge cell migration through dense matrix [39,40]. When nuclear integrity is compromised, exonucleases and DNA-repair pathways modulate how much nuclear material becomes available to innate immune sensors, and persistent envelope rupture can drive invasion-like inflammatory programs [41]. Activated fibroblasts also display nuclear mechanosensing and chromatin remodeling that may persist after the original mechanical cue changes [42]. The wound implication is that mechanical offloading, matrix softening, and cytoskeletal modulation may influence inflammatory state by reducing nuclear damage, not just by lowering gross tissue tension.

**Figure 1:**
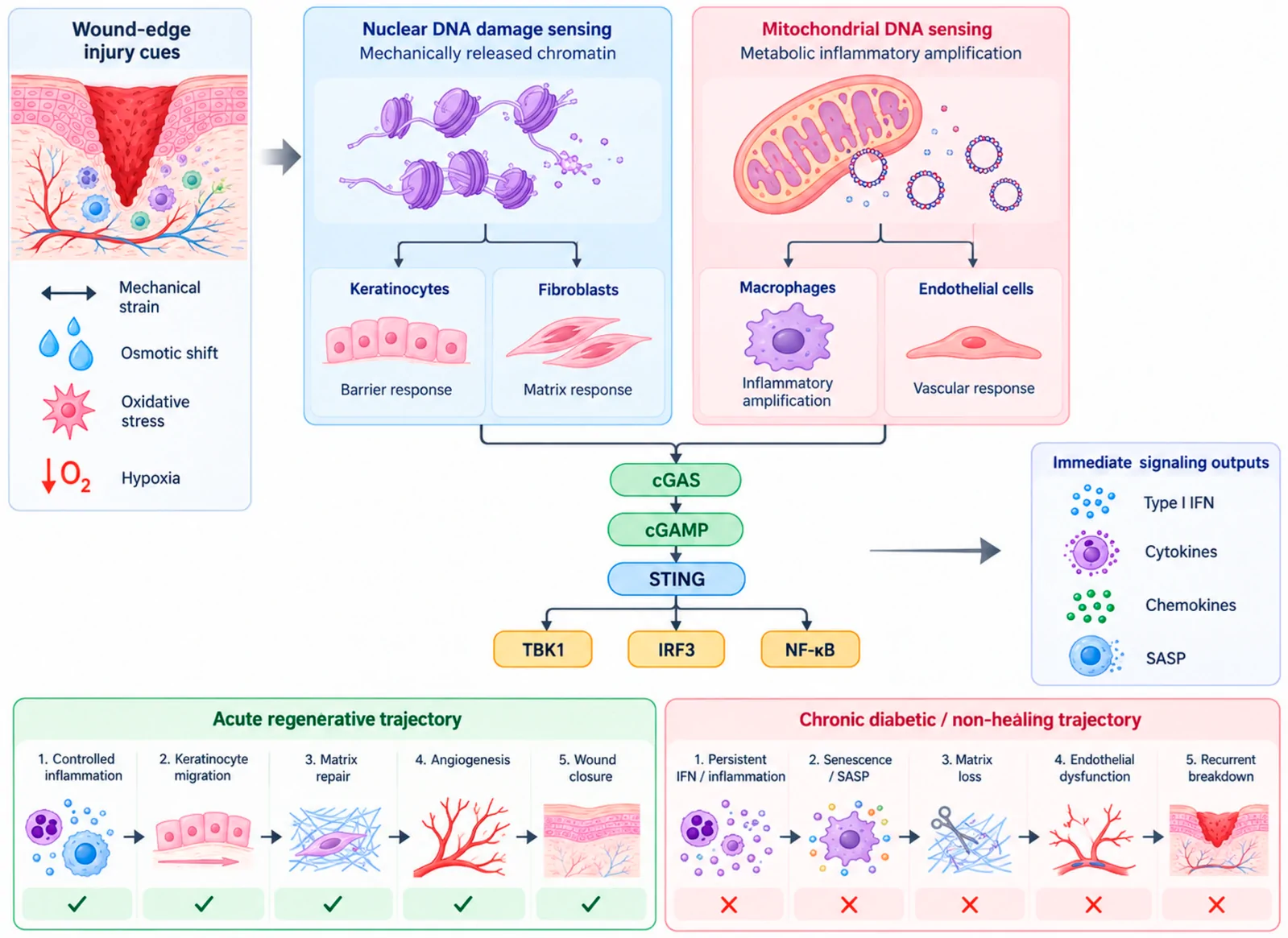
Nuclear damage-response code at the wound edge. Mechanical strain, osmotic shifts, oxidative/metabolic stress, nuclear chromatin exposure, mitochondrial DNA leakage, and cGAS–STING activation are integrated as a coupled injury-sensing circuit at the wound edge. The schematic distinguishes mechanically released nuclear DNA signals, emphasized in keratinocyte barrier/migration responses and fibroblast matrix remodeling, from mitochondrial DNA leakage, emphasized as a metabolically sensitive inflammatory amplifier in macrophage and endothelial compartments. Downstream signaling converges on type I interferon, cytokine, chemokine, and senescence-associated outputs, producing either a transient regenerative trajectory with matrix repair, angiogenesis, and wound closure or a persistent diabetic/non-healing trajectory characterized by chronic inflammation, senescence, matrix loss, endothelial dysfunction, and recurrent tissue breakdown.

Nuclear mechanotransduction provides a bridge between the classic scar literature and modern immune sensing. Lamins, chromatin organization, and nuclear-cytoskeletal coupling influence both structural resilience and gene accessibility; abnormalities in these systems are associated with nuclear fragility and disease phenotypes [43]. In acute wounds, some nuclear deformation is probably indispensable because collective epithelial migration and fibroblast invasion require cells to interpret force direction and spatial opportunity. In chronic wounds, however, the same system is challenged by a stiff scar-like matrix, repeated debridement, high glucose, oxidants, biofilm products, and abnormal edema. This creates a plausible sequence: matrix stiffness increases cytoskeletal tension; tension deforms nuclei and alters chromatin; nuclear rupture or chromatin leakage activates DNA-sensing pathways; inflammatory products stiffen and degrade matrix; and the cycle repeats. Figure 1 summarizes this model as a damage-response code rather than a one-directional pathway. It also highlights why reducing stiffness or oxidative stress may be ineffective unless paired with interventions that restore clearance and prevent reactivation. Within this model, keratinocytes are expected to weight nuclear-envelope damage toward migration and differentiation decisions, whereas fibroblasts are expected to weight it toward matrix remodeling, contractility, and chromatin-memory programs; mitochondrial DNA is treated separately as a metabolically sensitive amplifier of innate immune signaling. This proposed sequence remains a testable hypothesis for wounded skin and should be evaluated with paired measurements of matrix mechanics, nuclear-envelope rupture, cytosolic DNA sensing, and cell-type-resolved outcomes.

## 4. cGAS-STING as a Decision Node

Cytosolic DNA sensing gives the nuclear damage-response code its immunological amplifier. Cyclic guanosine monophosphate-adenosine monophosphate synthase detects cytosolic DNA and produces cyclic guanosine monophosphate-adenosine monophosphate, which activates stimulator of interferon genes and downstream interferon-regulatory and nuclear factor pathways [44,45]. Reviews of the pathway emphasize that it is no longer a virus-only defense module; it responds to nuclear DNA, micronuclei, mitochondrial DNA, chromatin fragments, and multiple forms of cellular stress [46,47,48,49]. DNA damage can therefore become inflammation when chromatin or micronuclear contents are mislocalized, and the cGAS-cGAMP-STING axis links genome instability to interferon and senescence programs [50,51,52]. In wound biology, this matters because tissue disruption generates physical and metabolic conditions that can expose DNA at the same time that cells must coordinate immunity and repair.

Senescence is a major route through which nuclear stress can become a non-healing wound program. Cytoplasmic chromatin fragments trigger inflammatory signaling in senescent cells, and innate sensing of those fragments through cyclic guanosine monophosphate-adenosine monophosphate synthase promotes senescence-associated inflammatory output [53,54]. Mitochondrial DNA adds a second source of danger-associated nucleic acid. Apoptotic caspases normally suppress mitochondrial DNA-induced stimulator-of-interferon-genes signaling, but mitochondrial outer membrane permeabilization, oxidant injury, and defective quality control can increase mitochondrial DNA efflux [55,56,57,58]. Autophagy also determines whether mitochondrial DNA becomes immunogenic or remains cleared, as shown by radiation-response studies and broader mitochondrial inflammatory biology [59]. The chronic wound hypothesis that follows is specific: diabetic oxidative stress and impaired clearance may feed cGAS-STING not only from damaged nuclei but also from injured mitochondria, creating a combined nuclear-mitochondrial danger field.

**Figure 2:**
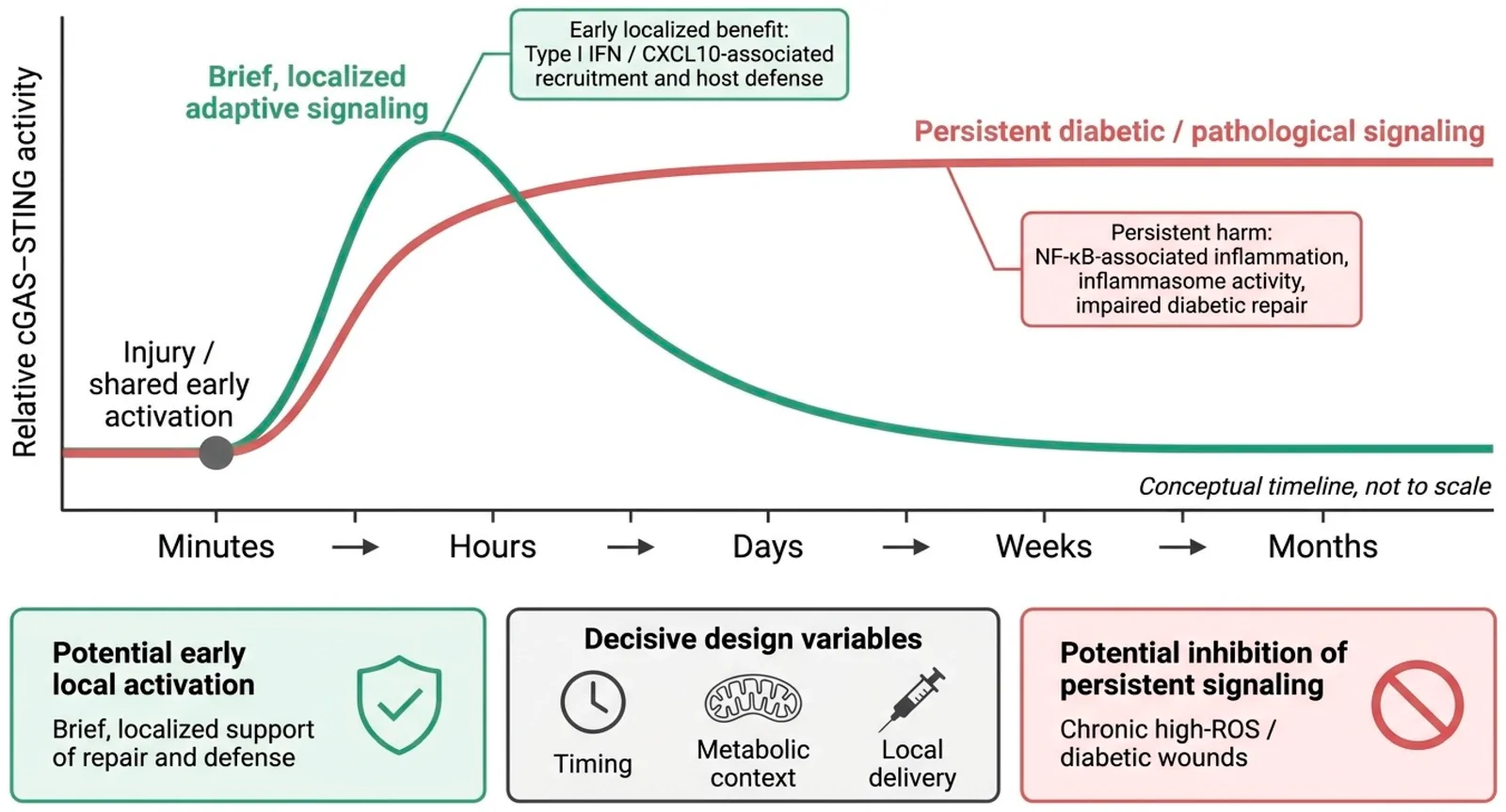
Temporal polarity of cGAS-STING in wound repair. The same pathway may support early chemotaxis and host defense when it is brief and localized, but impair diabetic repair when persistent. The schematic emphasizes timing, metabolic context, and local delivery as decisive design variables.

Direct wound studies show why cGAS-STING cannot be labeled simply good or bad. Topical activation of stimulator of interferon genes with cyclic guanosine monophosphate-adenosine monophosphate accelerated early skin wound repair in a mouse model through type I interferon and CXCL10-CXCR3-related mechanisms [60]. In diabetic skin, however, stimulator-of-interferon-genes activation promoted inflammatory responses and delayed healing, whereas inhibition improved closure in preclinical models [61]. High glucose triggered mitochondrial DNA-cGAS-STING signaling in macrophages, driving M1-like polarization and endothelial dysfunction in diabetic wound settings [62]. High glucose also activated endothelial STING and impaired angiogenesis, with pharmacological inhibition improving diabetic wound repair [63]. A diabetes-focused review similarly identifies cGAS-STING as a therapeutic target while emphasizing complications, context, and unresolved cell-type specificity [64]. Figure 2 therefore presents a temporal polarity model: brief activation may be useful early, but persistent activation is pathogenic in metabolically stressed wounds.

The most actionable insight is that cGAS-STING is embedded in trafficking and clearance networks. Autophagy induction through stimulator-of-interferon-genes trafficking is described as a primordial function of the pathway, suggesting that immune activation and cytoplasmic cleanup are historically linked rather than opposing processes [65]. Mammalian autophagy controls damaged organelles, protein aggregates, intracellular pathogens, and inflammatory tone, and its disruption is a plausible reason why diabetic wounds accumulate danger signals [66]. Autophagy also intersects with immunometabolism because phagocytes use quality-control pathways to decide whether they will clear debris, present inflammatory signals, or sustain cytokine production [67]. For wound therapy, this means that simple blockade of cGAS-STING could help a chronic diabetic ulcer but impair early antimicrobial defense or epithelial recruitment if delivered at the wrong time. Signal-gated therapy should instead inhibit persistent DNA-sensing only after enough debris clearance and host defense have occurred.

## 5. Senescence, Macrophages, and Failed Resolution

Cellular senescence is paradoxical in wound repair because a transient senescent program can be beneficial while persistent senescence can be destructive. Senescent cells support optimal repair in experimental wounds through platelet-derived growth factor-AA secretion, showing that cell-cycle arrest is not automatically harmful during acute repair [68]. Senescence-associated secretory phenotypes can also induce tissue plasticity and regenerative features under specific conditions [69]. Tissue damage and senescence provide signals for in vivo cellular reprogramming, reinforcing the idea that injury-induced plasticity requires transient stress states rather than complete suppression of damage responses [70]. Yet senescent cells change metabolic and secretory behavior over time, and chronic senescence is associated with inflammatory, proteolytic, and matrix-disruptive states [71,72]. Human diabetic foot ulcer network analyses reveal upregulation of senescence-associated markers and altered protein-interaction networks, supporting the clinical relevance of persistent senescence in stalled wounds [73].

Macrophages are the major interpreters of the wound damage field. They clear dead cells, orchestrate matrix remodeling, promote angiogenesis, and can also drive fibrosis and chronic inflammation [74,75]. A macrophage-pericyte axis can direct tissue restoration through amphiregulin-induced transforming growth factor beta activation, which illustrates how macrophages regulate stromal behavior rather than merely secrete inflammatory cytokines [76]. Skin-repair experiments also show that macrophages support myofibroblast proliferation and heterogeneity, and that CD301b-positive macrophages are required for effective skin healing [31,77]. In the nuclear damage-response model, macrophages determine whether DNA-rich debris becomes an inflammatory stimulus or a cleared substrate. Efferocytosis reviews emphasize that efficient recognition and engulfment of dying cells actively promote resolution, while failed clearance converts dead-cell cargo into secondary necrosis and inflammatory amplification [78,79].

Neutrophils add a chromatin-specific complication. Diabetes primes neutrophils to undergo neutrophil extracellular trap formation, and that chromatin externalization impairs wound healing in experimental and human contexts [80]. Neutrophil extracellular traps are built from decondensed chromatin and antimicrobial proteins; their biology is therefore directly relevant to cytosolic DNA-sensing and wound protease burden [81]. Although neutrophil extracellular traps evolved as host-defense structures, they can drive noninfectious inflammation, thrombosis, and tissue injury when not resolved [82]. In chronic wounds, extracellular chromatin may therefore act in three overlapping ways: it increases viscosity and biofilm compatibility, exposes histones and granule enzymes that damage tissue, and supplies nucleic-acid ligands that could sustain innate sensing. A nucleus-aware review must include neutrophils because persistent DNA in the wound bed is not produced only by ruptured nuclei or mitochondria inside resident cells; it is also exported deliberately by immune cells during dysregulated defense.

## 6. Cell-Type Circuits: Keratinocytes, Fibroblasts, Endothelium, and Microbiome

Keratinocytes are the most visible cells in closure, but their failure in chronic wounds is not simply slow migration. Single-cell epidermal profiling identifies discrete basal and differentiation states during homeostasis and repair, implying that wound-edge epidermis chooses between migration, proliferation, inflammation, and differentiation programs [12]. Diabetic foot ulcer single-cell work has now identified impaired epidermal differentiation, diabetes-associated keratinocyte states, and inflammatory ligand-receptor networks that may hold epidermis in an inactive or misdirected state [16]. Mechanistically, keratinocytes are exposed to high glucose, microbial products, reactive oxygen species, and matrix discontinuity, all of which can increase nuclear stress. If keratinocyte nuclei interpret this environment as persistent danger, then re-epithelialization may be blocked not because keratinocytes lack growth signals, but because differentiation and barrier restoration are overridden by inflammatory memory. This explains why therapies that add growth factors can disappoint when the underlying state classification remains chronic.

Fibroblasts translate nuclear and mechanical history into matrix architecture. Fibroblast heterogeneity in murine wounds, human skin, and diabetic ulcers shows that stromal populations differ in inflammatory signaling, matrix production, regenerative potential, and anatomical memory [11,13,14,15]. Regenerative outcomes require more than collagen deposition; they require fibroblasts to leave a contractile state, rebuild appropriate matrix, and avoid locking tissue into scar stiffness. Engrailed-1-positive fibroblast activation promotes scarring, whereas blocking that activation can yield wound regeneration without scarring in experimental settings [19]. Lef1-positive or other developmentally primed fibroblast states can preserve regenerative capacity, and multi-omic comparisons show that scarring and regenerative trajectories diverge at molecular levels before gross morphology reveals the outcome [18,20,21]. In the nuclear damage-response model, fibroblast nuclei are both sensors and recorders of matrix stiffness, with lamin content, chromatin organization, and mechanosensitive transcription contributing to persistent myofibroblast behavior.

**Figure 3:**
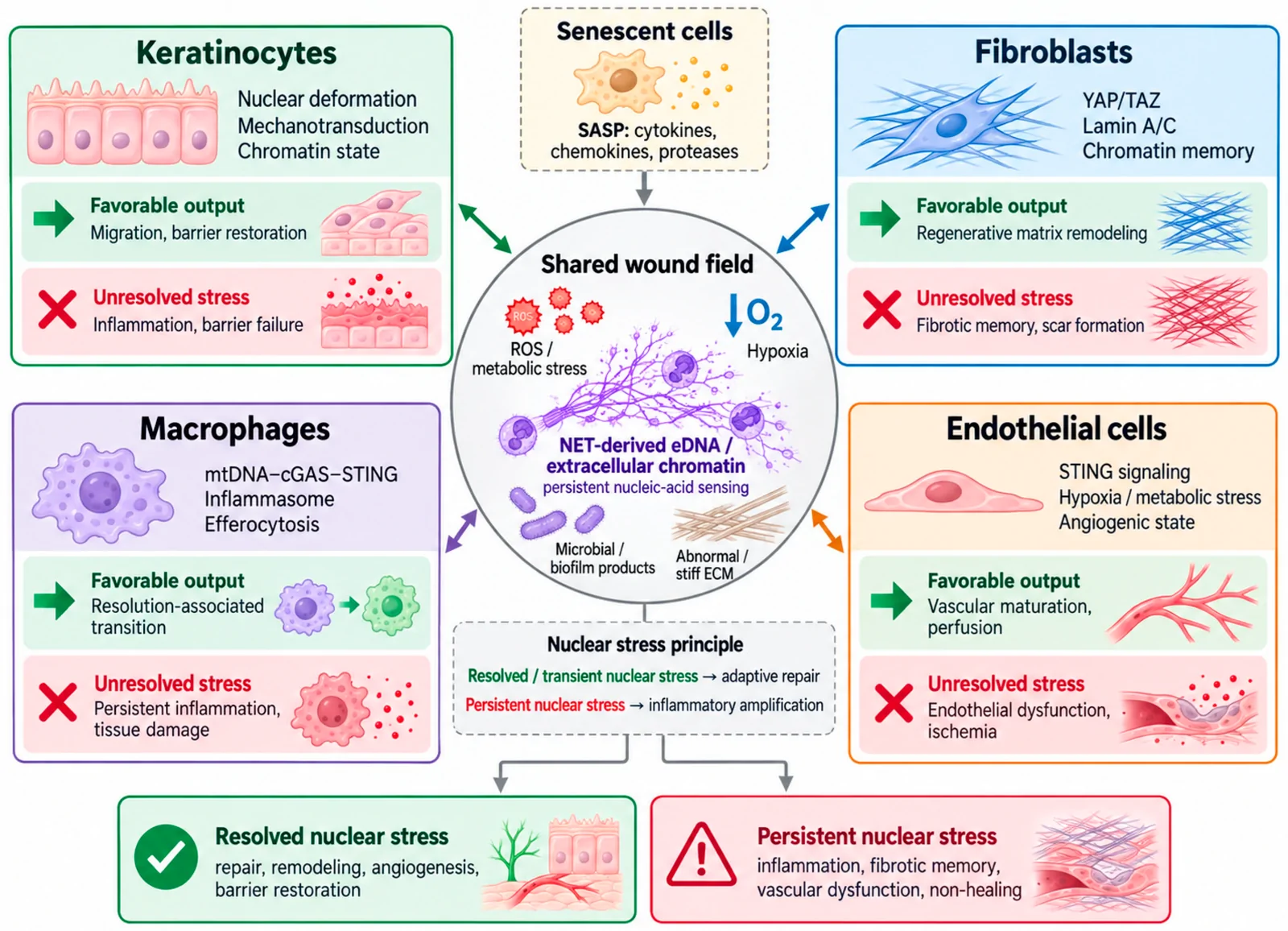
Cell-type circuitry linking nuclear stress to repair outcomes. Keratinocytes, fibroblasts, macrophages, and endothelial cells exchange signals with a shared wound field containing reactive oxygen species, hypoxia, abnormal or stiff extracellular matrix, microbial/biofilm products, and extracellular DNA. NET-derived extracellular DNA is emphasized as a persistent wound-field input that can sustain nucleic-acid sensing, protease burden, and biofilm-compatible inflammation. Repair outcome depends on whether nuclear stress is resolved, supporting migration, matrix remodeling, angiogenesis, and barrier restoration, or remains persistent, promoting inflammatory amplification, fibrotic memory, vascular dysfunction, and non-healing.

Endothelial cells and angiogenesis define whether a wound receives enough oxygen, nutrients, immune trafficking, and waste removal to progress. Diabetes is strongly associated with impaired wound angiogenesis, and angiogenic repair requires a balance between vessel growth and resolution rather than uncontrolled vascular stimulation [83,84]. Hypoxia-inducible pathways are central to wound repair, but chronic hypoxia can also select for inflammatory and proteolytic programs instead of orderly vascular maturation [85]. Toll-like receptor signaling and innate immune activation influence acute wound responses, connecting host-defense recognition to vascular and epithelial outcomes [86]. The emerging STING data sharpen this framework because high glucose can activate endothelial STING and impair angiogenesis, while inhibition reverses key endothelial defects [63]. Thus, nuclear or mitochondrial DNA sensing may be one reason that chronic diabetic wounds remain poorly vascularized despite abundant inflammatory signaling. Figure 3 summarizes these cell-type circuits and shows why no single cell type should be treated as the sole bottleneck.

The microbiome contributes to the same decision field by altering immune tone, redox stress, and extracellular DNA burden. Reviews of nonhealing diabetic wounds emphasize that wound microbial communities are not simply contaminants; they interact with host immunity and therapy response [87]. Temporal stability of chronic wound microbiota is associated with poor healing, suggesting that non-healing wounds can become ecologically entrenched [88]. Strain- and species-level variation in diabetic wound microbiomes correlates with clinical outcomes and therapeutic efficacy, making microbial identity relevant to host-state transitions [89]. Patient genetics also shapes chronic wound microbiome composition and healing, indicating that host and microbial programs co-evolve in the wound bed [90]. The nuclear damage-response code therefore has a microbial component: persistent microbes and biofilm matrix can maintain oxidant and protease stress, while extracellular microbial and host DNA can stabilize the very inflammatory environment that prevents epidermal differentiation and vascular recovery.

## 7. From Acute Signaling to Chronic Loops

The difference between regeneration, scarring, and chronicity may lie less in which pathway is present than in whether it resolves. Fibrosis reviews emphasize that persistent macrophage activation, myofibroblast contraction, extracellular matrix accumulation, and altered mechanotransduction reinforce one another across organs [91]. In skin, lineage-tracing studies identify dermal populations with intrinsic fibrogenic potential, supporting the idea that scar formation is partly a programmed cellular choice rather than only an excess-healing accident [92]. Chronic wounds represent a related but distinct failure: matrix is both degraded and stiffened, inflammation is both excessive and ineffective, angiogenesis is both stimulated and inadequate, and keratinocytes are both activated and unable to complete differentiation. A nuclear damage-response lens explains these contradictions because the same cells can receive mixed signals: close the wound, defend against danger, remain senescent, migrate through stiff matrix, and tolerate hypoxia simultaneously.

A practical model is to treat the wound as a classifier. During acute repair, damage signals should be high in amplitude but short in duration, spatially concentrated at the leading edge, and followed by autophagy, efferocytosis, vascular ingrowth, microbial control, and matrix remodeling. During chronicity, danger signals are lower or fluctuating but persistent, spread across the wound bed, and amplified by high glucose, biofilm, senescence, mitochondrial dysfunction, and mechanical memory. This distinction clarifies why the cGAS-STING literature appears contradictory: early activation can accelerate normal wound repair, whereas diabetic activation inhibits macrophage transition, endothelial function, and closure [60,61,62,63]. It also clarifies why senescence can support acute repair yet harm diabetic wounds [68,73]. The key therapeutic task is therefore not universal activation or inhibition; it is to reset signal duration, location, and cell-type specificity.

## 8. Biomaterials and Local Delivery

Smart wound dressings provide a route to manipulate the nuclear damage-response code locally, avoiding systemic immunosuppression. Modern smart dressings can sense wound chemistry, deliver drugs, modulate hydration, and respond to infection or oxidative cues [93]. Conductive biomaterials and electroactive dressings are particularly relevant because endogenous wound electric fields, ion flux, and mechanotransduction influence epithelial migration, cell polarity, and tissue organization [94]. Degradable conductive injectable hydrogels have been developed with antibacterial and antioxidative functions, illustrating how material properties can be aligned with inflammation control and tissue repair [95]. A nucleus-aware interface would extend these ideas by including nuclear-stress readouts, such as persistent interferon signatures, senescence-associated markers, mitochondrial DNA release, impedance changes consistent with epithelial barrier failure, or protease profiles that indicate unresolved inflammation.

**Figure 4:**
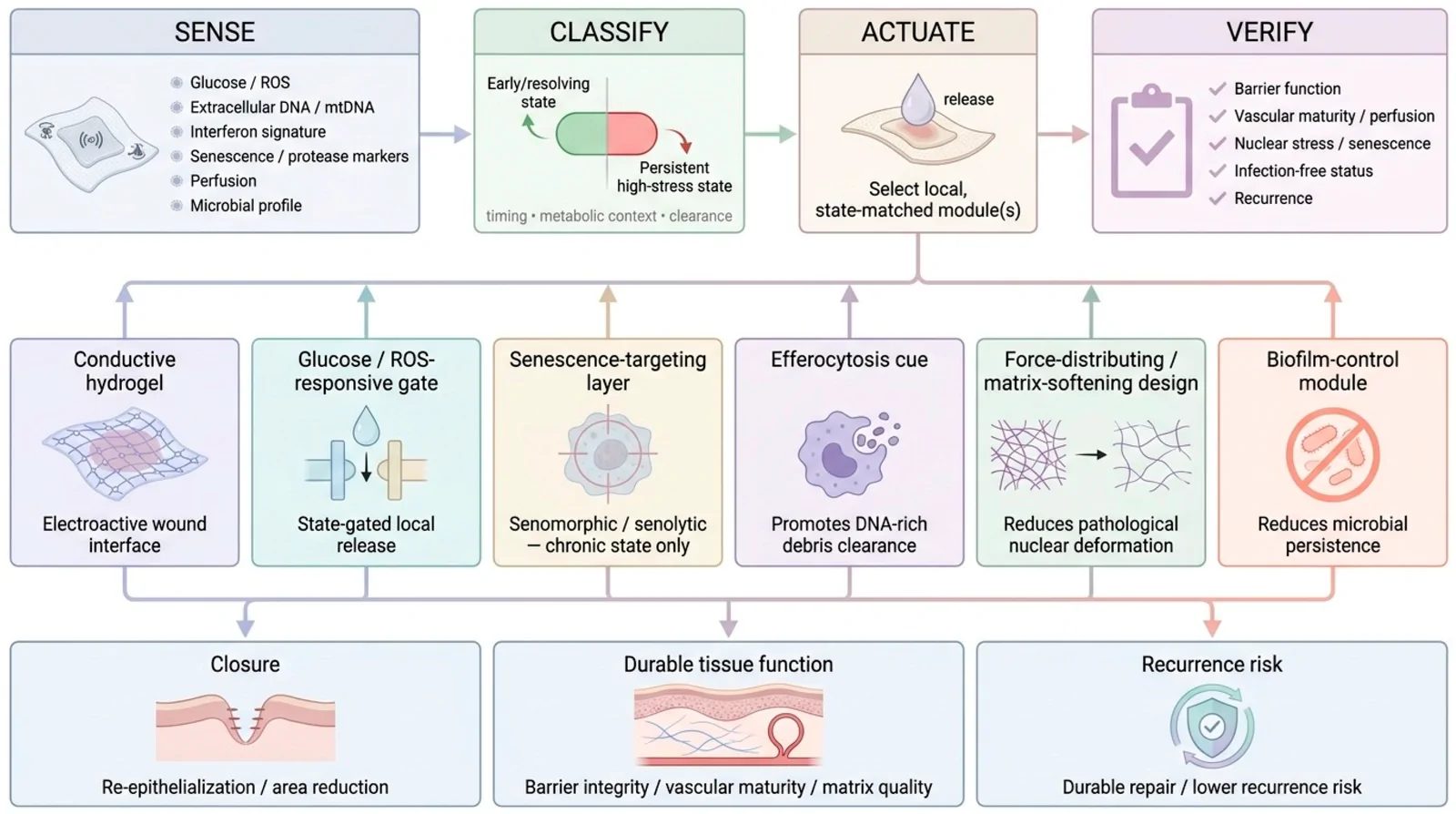
Translational design of nucleus-aware regenerative interfaces. A sense-classify-actuate-verify architecture is proposed for smart dressings and local therapies. The design separates wound closure from durable tissue quality and recurrence prevention.

Material design should also prevent a common translational error: closing the wound surface while leaving a recurrence-prone field beneath it. Hydrogel release systems that respond to glucose or reactive oxygen species could deliver STING inhibitors, antioxidants, or senomorphic drugs only when diabetic chemistry is present, while permitting early defense in clean acute wounds. Matrix-softening or force-distributing designs could lower nuclear deformation in fibroblasts and keratinocytes, reducing the mechanical input that feeds chromatin damage and fibrotic memory. Efferocytosis-promoting cues could help macrophages clear DNA-rich debris before strong anti-inflammatory therapy is applied, and biofilm modules could reduce microbial persistence without broadly suppressing host immunity. Figure 4 converts these principles into a sense-classify-actuate-verify architecture. The verification step matters because wound area reduction alone does not prove that nuclear stress, senescence, or endothelial dysfunction has resolved.

Safety constraints are equally important for adaptive dressings. Excessive matrix softening could weaken fragile peri-wound tissue, reduce tensile competence, or interfere with the contractile phase needed for early closure, especially in elderly, ischemic, or steroid-exposed skin. Local senomorphic or senolytic delivery should also be treated cautiously in infected wounds, because premature suppression or depletion of stress-responsive cells could impair antimicrobial defense, debris clearance, and granulation. These interventions therefore require dose-limited, spatially confined delivery and safety endpoints that include infection recurrence, local tissue fragility, delayed granulation, and recurrence after closure.

## 9. Clinical Translation and Trial Design

Clinical studies of nucleus-aware wound therapy should stratify wounds by state rather than by diagnosis alone. Diabetic foot ulcer, venous leg ulcer, and pressure injury labels remain necessary, but each category likely contains wounds with different mixtures of hypoxia, senescence, biofilm, nuclear rupture, mitochondrial stress, and defective clearance. Recent single-cell and bulk transcriptomic analyses of diabetic foot ulcers offer templates for identifying healing-associated fibroblast and macrophage states, epidermal differentiation blocks, and non-healing mechanisms [15,16,96]. These approaches can be paired with conventional wound measurements, perfusion testing, microbial profiling, and histology. Table 2 proposes translational readouts that separate closure speed from repair quality. The most important outcome should be durable, functional tissue, not simply a smaller wound at week 12. Biomarkers should therefore be collected longitudinally to determine whether a therapy compresses the chronic damage-response loop or only suppresses one visible output. Clinical deployment also requires attention to assay feasibility: wound-bed DNA, nuclear rupture, phospho-TBK1/IRF3, senescence, or extracellular chromatin markers may require tissue biopsy, specialized immunostaining, or multiplex molecular assays, which can increase cost, complicate repeated sampling, and limit inter-laboratory standardization.

Trial timing should reflect the temporal polarity model. Acute traumatic or surgical wounds may benefit from short-lived activation of host-defense and chemotactic modules, whereas chronic diabetic wounds with high oxidative stress may require inhibition of persistent STING, reduction in senescence-associated secretory phenotype burden, or restoration of autophagic and efferocytic clearance. Safety monitoring should include infection recurrence, delayed granulation, excessive scarring, and systemic inflammatory markers because DNA-sensing pathways participate in host defense. The most informative early trials would use adaptive local delivery rather than fixed systemic dosing. They would test whether biomarker-defined chronic wounds convert toward a resolving state: reduced interferon and inflammasome signatures, improved keratinocyte differentiation, improved endothelial markers, lower extracellular DNA or protease burden, and restoration of macrophage clearance programs. This design accepts that the same target can be harmful in one wound state and necessary in another.

The gap between preclinical promise and clinical implementation is likely to be widest for markers or interventions validated only in controlled experimental wounds. Clinical ulcers are heterogeneous, recurrent, polymicrobial, ischemic, and exposed to variable offloading and debridement. Early clinical studies should therefore report biomarker analytical validity, biological variability, sampling burden, cost, and the relationship between biomarker change, closure durability, infection-free survival, and patient-centered outcomes.

Four recent developments strengthen the feasibility of the proposed translational program. Mechanotransduction-focused scar literature now treats force-sensitive signaling as an actionable target rather than as a descriptive wound feature, which supports testing nuclear-deformation readouts alongside offloading and matrix-softening interventions [97]. Senescence-focused wound reviews emphasize that p16, p21, SASP mediators, and tissue context must be interpreted together because senescence can be reparative during acute closure but harmful when it persists in aged or diabetic skin [98]. Delivery studies also show that local anti-inflammatory biomaterials can move beyond passive coverage; for example, disulfiram-loaded nanovesicle hydrogels reduced NET-linked inflammatory outputs and promoted angiogenesis and re-epithelialization in diabetic wound models [99]. Finally, human single-cell atlases continue to uncover wound-state-specific fibroblast programs, including APOE-positive pro-inflammatory fibroblasts in diabetic foot ulcers, providing a route for matching mechanistic therapies to patient-level wound biology [100].

## 10. Limitations

This Perspective has several limitations. It is a narrative synthesis rather than a systematic review or meta-analysis; therefore, it prioritizes mechanistic coherence and hypothesis generation over pooled effect estimates. Some elements of the proposed nuclear damage-response code are directly supported by wound studies, whereas others are extrapolated from mechanobiology, cancer-cell migration, mitochondrial immunology, senescence, and fibrosis models. The framework should consequently be tested with cell-type-resolved human wound sampling, longitudinal biomarker validation, and intervention studies that distinguish transient repair-supportive stress signaling from persistent pathogenic activation.

## 11. Conclusions

Cutaneous wound repair is not governed only by inflammatory mediators, growth factors, microbes, or matrix components considered separately. The evidence reviewed here supports a more integrated model in which the nucleus functions as a damage integrator that converts mechanical strain, chromatin mislocalization, mitochondrial injury, and unresolved debris into transcriptional and innate immune decisions. This model explains several otherwise discordant observations: inflammation can be required for repair yet destructive in diabetes; senescence can promote acute closure yet sustain chronic ulcers; fibroblasts can regenerate appendage-supportive matrix or produce scar; and STING activation can accelerate normal repair but impair diabetic healing. The model also creates a translational path. Instead of asking whether a pathway should be turned on or off, future therapies should ask whether the signal is brief or persistent, local or diffuse, cleared or accumulating, regenerative or scar-forming. The next generation of wound dressings and trials should therefore be state-aware, locally adaptive, and judged by durable restoration of tissue function.

## Figures and Tables

**Table 1:** Mechanistic nodes in the proposed nuclear damage-response code.

Mechanistic Node	Evidence Anchor	Repair Interpretation	Testable Prediction
Nuclear deformation	Nucleus changes shape under spatial constraint and cell migration	Useful when it orients migration; harmful when it produces repeated envelope stress	Offloading or matrix-softening should reduce interferon and senescence signatures in stiff wound edges
Nuclear envelope rupture	Rupture exposes chromatin and requires ESCRT-mediated repair	A physical event can become an inflammatory trigger	Persistent wounds should show higher nuclear rupture markers than matched acute wound edges
Cytosolic DNA sensing	cGAS-STING responds to nuclear and mitochondrial DNA	Converts unresolved cell stress into inflammatory transcription	Localized STING inhibition should help high-ROS diabetic wounds more than clean acute wounds
Autophagy and mitophagy	Autophagy limits damaged organelles and regulates STING trafficking	Clearance capacity determines whether DNA remains immunogenic	Autophagy rescue should lower mtDNA-STING output before closure visibly accelerates
Senescence-associated secretion	Senescent cells can support acute repair but accumulate in diabetic ulcers	Timing decides whether senescence is regenerative or chronic	Senomorphic therapy should be beneficial only after early repair signals have occurred
Macrophage efferocytosis	Efficient clearance of dying cells actively resolves inflammation	Macrophages decide whether DNA-rich debris is cleared or amplified	Efferocytosis markers should predict response to anti-inflammatory biomaterials

Abbreviations: mtDNA, mitochondrial DNA; ROS, reactive oxygen species; STING, stimulator of interferon genes.

**Table 2:** Translational decisions for nucleus-aware wound trials.

Translational Decision	Candidate Readouts	Therapeutic Implication	Risk to Monitor
Classify wound state	Interferon response genes, extracellular DNA, senescence markers, protease activity, perfusion, microbial profile; assay cost, biopsy or wound-fluid sampling feasibility, inter-laboratory reproducibility	Select activation for clean early wounds or inhibition/resolution for chronic diabetic wounds	Misclassification may suppress host defense or prolong inflammation; impractical biomarkers may prevent adoption
Reset mechanical memory	Matrix stiffness, collagen alignment, lamin A/C, YAP/TAZ localization, fibroblast contractility	Use offloading, matrix-softening materials, or force-distributing dressings	Over-softening may impair contraction and tensile strength; peri-wound fragility and delayed tensile recovery should be monitored
Restore clearance	Efferocytosis markers, autophagy flux markers, mitochondrial DNA, apoptotic-cell burden	Deliver pro-efferocytic and autophagy-supportive cues before broad cytokine suppression	Excess clearance stimulation may mask infection or necrosis
Target persistent DNA sensing	STING, phosphorylated TBK1/IRF3, type I interferon genes, NF-kB and inflammasome outputs	Use local signal-gated cGAS-STING inhibition in metabolically stressed chronic wounds	Early inhibition may slow chemotaxis, antimicrobial defense, or epithelial recruitment
Control senescence burden	p16, p21, SASP cytokines, MMPs, IGFBP family markers, cell-cycle arrest signatures	Use senomorphic or senolytic layers after defining chronic senescence state	Aggressive senolysis may remove transient repair-supportive cells; local delivery requires infection-aware dosing and follow-up
Verify repair quality	Barrier function, recurrence, scar pliability, vascular maturity, pain, infection-free survival	Move beyond percentage wound-area reduction as a sole endpoint	A closed but fragile epithelial surface may recur quickly

Abbreviations: cGAS, cyclic guanosine monophosphate-adenosine monophosphate synthase; SASP, senescence-associated secretory phenotype; STING, stimulator of interferon genes; TBK1, TANK-binding kinase 1.

## Data Availability

No new data were created or analyzed in this study. Data sharing is not applicable to this article.

## Abbreviations

cGAS, cyclic guanosine monophosphate-adenosine monophosphate synthase; cGAMP, cyclic guanosine monophosphate-adenosine monophosphate; CXCL, C-X-C motif chemokine ligand; DNA, deoxyribonucleic acid; ESCRT, endosomal sorting complexes required for transport; HIF, hypoxia-inducible factor; IRF, interferon regulatory factor; LINC, linker of nucleoskeleton and cytoskeleton; mtDNA, mitochondrial DNA; NET, neutrophil extracellular trap; NF-kB, nuclear factor kappa B; ROS, reactive oxygen species; SASP, senescence-associated secretory phenotype; STING, stimulator of interferon genes; TBK1, TANK-binding kinase 1; YAP/TAZ, Yes-associated protein/transcriptional coactivator with PDZ-binding motif.
